# Self-reported motivators for HIV testing in the treat-all era among HIV positive patients in Johannesburg, South Africa

**DOI:** 10.1097/MD.0000000000025286

**Published:** 2021-04-16

**Authors:** Idah Mokhele, Tembeka Sineke, Jonas Langa, Dorina Onoya

**Affiliations:** a Health Economics and Epidemiology Research Office, Department of Internal Medicine, School of Clinical Medicine, Faculty of Health Sciences, University of the Witwatersrand; b Right to Care, Johannesburg, South Africa.

**Keywords:** antiretroviral therapy, human immunodeficiency virus, HIV testing, motivators for HIV testing, South Africa

## Abstract

To explore associations between self-reported ill-health as a primary motivator for HIV-testing and socio-demographic factors.

Four local primary healthcare clinics in Johannesburg, South Africa.

A total of 529 newly HIV diagnosed adults (≥18 years) enrolled from October 2017 to August 2018, participated in the survey on the same day of diagnosis.

Testing out of own initiative or perceived HIV exposure was categorized as asymptomatic. Reporting ill-health as the main reason for testing was categorized as symptomatic. Modified Poisson regression was used to evaluate predictors of motivators for HIV testing.

Overall, 327/520 (62.9%) participants reported symptoms as the main motivator for testing. Among the asymptomatic, 17.1% reported potential HIV exposure as a reason for testing, while 20.0% just wanted to know their HIV status. Baseline predictors of symptom-related motivators for HIV testing include disclosing intention to test (aPR 1.4 for family/friend/others vs partners/spouse, 95% CI: 1.1–1.8; aPR 1.4 for not disclosing vs partners/spouse, 95% CI: 1.1–1.7), and HIV testing history (aPR 1.2 for last HIV test >12-months ago vs last test 12-months prior, 95% CI: 1.0–1.5; aPR 1.3 for never tested for HIV before vs last test 12-months prior, 95%CI:1.0–1.6).

Findings indicate that newly diagnosed HIV positive patients still enter care because of ill-health, not prevention purposes. Increasing early HIV testing remains essential to maximize the benefits of expanded ART access.

## 1 Introduction

The South African government has made a substantial investment to expand the national ART program. This includes adopting the World Health Organization recommended universal-test-and-treat (treat-all) strategy to initiate antiretroviral therapy (ART) as soon as individuals are diagnosed with HIV, regardless of CD4 cell count.^[1,2]^ This strategy is anticipated to increase access to ART and decrease losses to HIV care among newly diagnosed patients.^[3]^ The treat-all strategy is meant to complement HIV case-finding strategies reflected in the revised 2016 National HIV Testing Services (HTS) policy^[4]^ and are both supportive of goals set in the National Strategic Plan for HIV and STI prevention and treatment 2017–2022 and the UNAIDS 90–90–90 targets.^[5–7]^

HIV testing is essential for expanding treatment; it is the entry point into HIV care for people living with HIV. Early HIV diagnosis facilitates early HIV treatment uptake, which is associated with reduced likelihood of onward transmission, better response to ART, and reduced HIV related morbidity and mortality.^[8–10]^ Potential benefits to early ART access are reliant on the majority of healthy individuals seeking HIV testing services and taking up ART when diagnosed with HIV. South Africa has recently made great strides in expanding HIV treatment services by adopting a more comprehensive approach to HIV testing services. This includes expanding testing strategies to include provider-initiated counselling and testing (PICT), couple's counselling and testing, and home-based counselling and testing (HBCT), as well as the more recently adopted HIV self-testing (HIVST).^[4,11]^ These efforts have put South Africa on a positive trajectory to meeting the first 90% target of people living with HIV knowing their status.^[12]^

However, many individuals remain unaware of their HIV status in South Africa where over a million of those infected with HIV remain undiagnosed.^[13]^ It is expected that as more people with HIV are diagnosed, finding those undiagnosed will become progressively more challenging and resource intensive.^[14]^ Additionally, only two-thirds of those living with HIV are initiated on ART, which is still considerably below the second 90% target of those who know their positive HIV status being initiated on ART.^[6,12,15]^ Barriers to HIV testing previously identified include low HIV risk perceptions, fear of testing positive and, HIV related stigma (anticipated and internalized).^[16–19]^

Few studies have examined HIV-testing decision-making since the start of the treat-all era. Thus, we explored motivators for HIV testing in the treat-all era and associations between self-reported ill-health as a primary reason for testing and socio-demographic factors. As access to ART is expanded, understanding to what extent individuals are still motivated by the onset of symptoms to access HIV testing services is essential. Results can inform improved strategies for effective ART demand creation in the era of expanded ART access in South Africa.

## 2 Methods

### 2.1 Study design and populations

**Figure 1:**
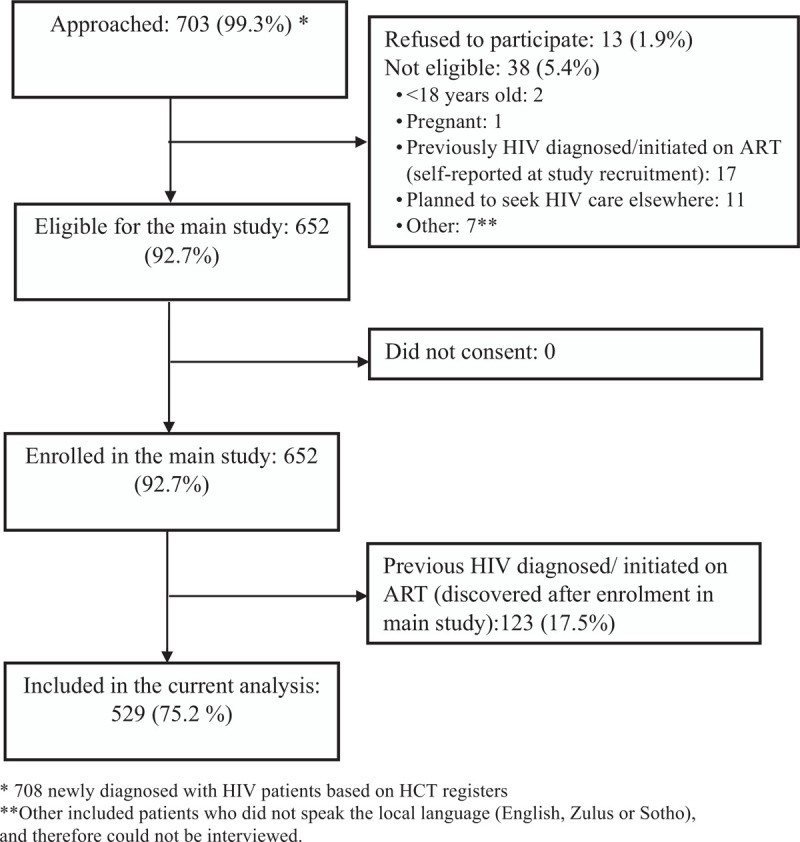
Patient recruitment, eligibility, and enrolment of adults (≥18 years) at 4 clinics in Johannesburg.

As part of a prospective study evaluating ART deferral among newly diagnosed HIV infected individuals at 4 participating local primary healthcare (PHC) clinics in Johannesburg, South Africa,^[20]^ a baseline cross-sectional survey was conducted among 652 participants from October 2017 to August 2018. Eligibility requirements for the study included being newly diagnosed of HIV on the day of study enrolment, being 18 years or older, not previously initiated on ART, not pregnant, not planning to get treatment elsewhere, physically and psychologically well enough to participate, and willing to provide informed consent. All participants provided written informed consent to participate in the study. Consent forms translated from English into Sotho and Zulu and administered in the participant's preferred language (English, Sotho or Zulu). Of the patients that were approached to participate in the main study (n = 703), 1.9% refused, while 5.4% were not eligible (Fig. 1). Further, a total of 123/652 (18.9%) of study participants enrolled in the main study were found to be known HIV positive patients but presented for testing as new patients. The current analysis includes cross-sectional data collected using a baseline questionnaire at study enrolment among 529 eligible participants.

### 2.2 Data collection

Eligible and consenting patients were recruited consecutively via referrals from PHC-based HIV lay counsellors and participated in the baseline survey on the same day they were diagnosed, after receiving their HIV test result. Participant sociodemographic, healthcare-seeking and sexual risk behavior, assessment of current psychosocial wellbeing, and social support were collected using a structured questionnaire administered in the participant's preferred language (English, Sotho or Zulu) by trained study interviewers.

### 2.3 Analytical variables

Reasons for HIV testing were assessed by a question asking the participant the main reason for coming to the clinic for an HIV test. Response choices were:

1. I was feeling ill,
2. I had sex without a condom,
3. I had sex with someone who is HIV positive,
4. I was sexually assaulted,
5. I have had many sexual partners,
6. My partner has been unfaithful,
7. My partner told me to get tested,
8. My partner was ill or died,
9. My child was ill or died,
10. I was taking care of someone with HIV,
11. I was offered a test by a health provider as routine part of care,
12. Other, (specify).

Patients reporting reasons related to risky sexual behavior or other potential HIV exposures were regarded to be motivated by current/previous HIV risk. Reasons provided under “Other” were categorized based on whether they were symptom or perceived HIV risk-related. The majority reported just wanting to know or just to check their HIV status, which we grouped into an additional category (just to know). We then dichotomized self-reported reasons for HIV testing into asymptomatic and symptomatic. Patients who reported testing out of their own initiative or because of a perceived HIV exposure, and those offered a test as part of routine care were categorized as asymptomatic.

We measured perceived social support (PSS) using an 8-item measure of social support where participants indicated the overall level of agreement with the support they have access to (Cronbach's alpha = 0.61).^[21]^ Rating of overall satisfaction for each item ranged from 1 to 4. Mean scores were categorized as either “low PSS” (score < 2), “medium (2 to < 3), or “high PSS” (score > = 3). We developed a household amenities index through factor analysis of participants’ household characteristics (type of toilet facilities, energy used for cooking, housing structure, household density, and food availability), and ownership of household assets (television, radio, refrigerator, satellite television, cellular telephone, landline telephone, microwave oven, and personal computer).^[22]^ The total score for the household amenities index ranged from 0 to 1, with higher total scores reflecting greater household access to amenities (Cronbach alpha = 0.81). A cut-off score of 0.3 or less indicated “low” amenities score, above 0.3 to 0.67 indicated “medium” amenities score, and a score higher than 0.67 indicated “high” amenities score.

Depression was measured using the Centre for Epidemiologic studies-Depression (CES-D) 10 scale, a 10-item questionnaire with a four-point scale (scores range 0 to 3) that measures general depressive symptoms experienced up to 7 days prior.^[23,24]^ The total score ranged from 0 to 30 with higher scores reflecting greater occurrence of depression (Cronbach alpha = 0.80), with a cut-off score of 12 or higher indicating the presence of major depressive symptoms.^[24,25]^ We created a dichotomous variable for depression categorized into no depression (CES-D 10 total score <12) and major depressive symptoms (CES-D 10 total score ≥12).

Other patient-level factors collected include sociodemographic characteristics: age, sex, highest education completed, English literacy, marital status; employment status, whether the patient is the household breadwinner, the number of child dependants and source of primary income. We assessed health care seeking behavior using history of visiting any other health provider or clinic, and HIV testing history. Factors relating to sexual risk behavior assessed included condom usage at last sex, and number of sexual partners in the preceding 12 months. Assessment of current social support factors included to whom the patients had disclosed their intention to come for HIV testing to, whether anyone accompanied them to the testing clinic and their intention to disclose their HIV status. Blood collection for baseline CD4 counts was done on the day of testing. Baseline CD4 results are categorized as <350, 350 to 500, and >500 cells/μl.

### 2.4 Statistical analysis

We used descriptive statistics to summarize participant characteristics at study enrolment. Continuous variables were described using medians and interquartile ranges (IQR) where appropriate. Categorical variables were described using frequencies and percentages. We accounted for missing data by including a “missing” category where more than 5% of the data were missing. Modified Poisson regression with robust standard errors was used to evaluate associations between baseline characteristics and the primary motivator for HIV testing. The Poisson model estimate adjusted prevalence ratio recommended for cross-sectional studies assessing binary outcomes with a prevalence greater than 10%.^[26,27]^ Factors identified with a univariate *P* value <.1 and a priori variables of importance such as sex and age were included in the adjusted model. Adjusted prevalence ratios (aPRs) with 95% confidence intervals (CIs) are presented.

Data analysis was conducted using STATA version 14 (StataCorp, College Station, TX).

### 2.5 Ethical review

The study was approved by the Human Research Ethics Committee (Medical) of the University of the Witwatersrand (Wits HREC M1704122). All personal identifiers were removed from the final analytic dataset.

## 3 Results

### 3.1 Baseline social and demographic characteristics

A total of 529 patients (plural) were included in the analysis (Table 1). Almost two-thirds were female (62.6%), and the median age at enrolment was 33 years (IQR 28.0–39.0). A total of 14.4% were married, with a high proportion of men (21.2%) married compared to women (10.3%). While 18.9% of study participants were not in a sexual relationship. A total of 39.5% had been living in their current house for more than 5 years, and for over a third (39.5%), this was their primary residence. The majority (86.2%) had at least a secondary school level education, and 56.2% had high English literacy. A higher proportion of females (60.8%) had high English literacy than males (48.5%). The majority of male participants (78.7%) were employed, and similar proportions (77.7%) were breadwinners of their households, whereas almost half of the female participants were dependent on others for financial support and only 36.8% were breadwinners. Overall, the majority (94.1%) reported their household access to basic amenities as medium to high.

**Table 1:** Participant sociodemographic characteristics (n = 529).

	Female		Male		Total	
	n	% (95% CI)	n		n	% (95% CI)
Age at HIV diagnosis, years Median (IQR)		32.0 (27.0–37.0)		36 (31.0–43.0)		33.0 (28.0–39.0)
18–29.99	129	39.0 (33.8–44.4)	39	19.7 (14.7–25.9)	168	31.8 (27.9–35.9)
30–39.99	145	43.8 (38.5–49.2)	91	46.0 (39.1–53.0)	236	44.6 (40.4–48.9)
40+	57	17.2 (13.5–21.7)	68	34.3 (28.0–41.3)	125	23.6 (20.2–27.4)
Marital status						
Married	34	10.3 (7.4–14.1)	42	21.2 (16.0–27.2)	76	14.4 (11.6–17.7)
In a relationship (living together)	121	36.7 (31.6–42.0)	71	35.9 (29.4–42.80	192	36.4 (32.5–40.6)
In a relationship (not living together)	107	32.4 (27.6–37.7)	53	26.8 (21.0–33.4)	160	30.3 (26.5–34.4)
Not in a relationship	68	20.6 (16.6–25.3)	32	16.2 (11.6–22.00	100	18.9 (15.8–22.5)
Highest education level						
Primary school or less	40	12.1 (9.0–16.1)	33	16.7 (12.1–22.60	73	13.8 (11.1–17.0)
Some secondary school	189	57.1 (51.7–62.3)	122	61.6 (54.6–68.2)	311	58.8 (54.5–62.9)
>=Grade 12	102	30.8 (26.1–36.0)	43	21.7 (16.5–28.0)	145	27.4 (23.8–31.4)
English literacy						
I can read very well	200	60.8 (55.4–65.9)	96	48.5 (41.6–55.5)	296	56.2 (51.9–60.4)
I can read somewhat	100	30.4 (25.6–35.6)	80	40.4 (33.8–47.4)	180	34.2 (30.2–38.3)
I cannot read	29	8.8 (6.2–12.4)	22	11.1 (7.4–16.3)	51	9.7 (7.4–12.5)
Employment status						
Employed	151	46.0 (40.7–51.5)	155	78.7 (72.4–83.9)	306	58.3 (54.0–62.4)
Unemployed	177	54.0 (48.5–59.3)	42	21.3 (16.1–27.6)	219	41.7 (37.6–46.0)
Primary source of income						
Paid job, salary or business	166	50.6 (45.2–56.0)	168	86.2 (80.5–90.4)	334	63.9 (59.6–67.9)
Spouse/ partner	85	25.9 (21.4–30.9)	9	4.6 (2.4–8.7)	94	18.0 (14.9–21.5)
Parents/ relatives/ friends/other	77	23.5 (19.2–28.4)	18	9.2 (5.9–14.2)	95	18.2 (15.1–21.7)
Breadwinner of household						
Yes	120	36.8 (31.7–42.2)	153	77.7 (71.3–83.0)	273	52.2 (47.9–56.5)
No	206	63.2 (57.8–68.3)	44	22.3 (17.0–28.7)	250	47.8 (43.5–52.1)
Access to basic necessities (amenities score)						
Low	13	4.1 (2.4–6.9)	17	8.9 (5.6–14.0)	30	5.9 (4.1–8.3)
Medium	127	39.7 (34.5–45.2)	77	40.5 (33.7–47.7)	204	40.0 (35.8–44.3)
High	180	56.3 (50.1–61.6)	96	50.5 (43.4–57.6)	276	54.1 (49.8–58.4)
Primary house						
Current house	124	38.2 (33.0–43.6)	82	41.8 (35.1–48.9)	206	39.5 (35.4–43.8)
Another province/rural	111	34.2 (29.2–39.5)	65	33.2 (26.9–40.1)	176	33.8 (29.8–38.0)
Another country	90	27.7 (23.7–32.8)	49	25.0 (19.4–31.6)	139	26.7 (23.0–30.7)
Duration at current house						
Less than 1 year	82	24.9 (20.5–29.9)	31	15.7 (11.3–21.5)	113	21.5 (18.2–25.2)
1–5 years	121	36.8 (31.7–42.1)	54	27.4 (21.6–34.10	175	33.3 (29.4–37.4)
More than 5 years	126	38.3 (33.2–43.7)	112	56.9 (49.9–63.6)	238	45.2 (41.0–49.5)
Live in						
Own home or rental	238	72.8 (67.7–77.3)	167	84.8 (79.0–89.2)	405	77.3 (73.5–80.7)
Friends/other's home	89	27.2 (22.7–32.3)	30	15.2 (10.8–21.0)	119	22.7 (19.3–26.5)
Lives with						
Partner/spouse	145	50.3 (44.6–56.1)	93	49.2 (42.1–56.3)	238	49.9 (45.4–54.4)
Family/friends	91	31.6 (26.5–37.2)	30	15.9 (11.3–21.8)	121	25.4 (21.7–29.5)
Alone	52	18.1 (14.0–23.0)	66	34.9 (28.4–42.0)	118	24.7 (21.1–28.8)
Number of child dependants						
None	177	53.5 (48.1–58.8)	135	68.2 (61.3–74.3)	312	59.0 (54.7–63.1)
1 child	67	20.2 (16.2–24.9)	28	14.1 (9.9–19.80	95	18.0 (14.9–21.5)
2 or more children	87	26.3 (21.8–31.3)	35	17.7 (12.9–23.7)	122	23.1 (19.7–26.9)
Recent clinic attendance (any)						
Never	36	11.1 (8.1–15.0)	57	28.9 (23.0–35.7)	93	17.8 (14.8–21.3)
within a year	193	59.4 (53.9–64.6)	69	35.0 (28.6–42.0)	262	50.2 (45.9–54.5)
More than a year ago	96	29.5 (24.8–34.7)	71	36.0 (29.6–43.0)	167	32.0 (28.1–36.1)
Number of sexual partners in the past 12 months						
None	33	10.2 (7.3–14.0)	13	6.6 (3.9–11.1)	46	8.8 (6.7–11.6)
1 Partner	204	63.2 (57.7–68.3)	97	49.2 (42.3–56.20	301	57.9 (53.6–62.1)
>=2 partners	86	26.6 (22.1–31.7)	87	44.2 (37.3–51.2)	173	33.3 (29.3–37.4)
Condom use at last sex						
Yes	97	29.8 (25.1–35.1)	68	34.5 (28.2–41.5)	165	31.6 (27.8–35.7)
No	228	70.2 (64.9–74.9)	129	65.5 (58.5–71.8)	357	68.4 (64.3–72.2)
Last HIV test before current test						
<=12 months prior	104	32.1 (27.2–37.4)	34	17.3 (12.6–23.2)	138	26.5 (22.9–30.5)
>12 months prior	161	49.7 (44.3–55.1)	70	35.5 (29.1–42.5)	231	44.3 (40.1–48.6)
Never tested for HIV before	59	18.2 (14.4–22.8)	93	47.2 (40.3–54.2)	152	29.2 (25.4–33.2)
Disclosed intention to test for HIV						
partner/spouse	105	32.4 (27.5–37.7)	82	41.6 (34.9–48.7)	187	35.9 (31.9–40.1)
Family/Friends/Other	99	30.6 (25.8–35.8)	32	16.2 (11.7–22.1)	131	25.1 (21.6–29.1)
No one	120	37.0 (31.9–42.5)	83	42.1 (35.4–49.2)	203	39.0 (34.9–43.2)
Person accompanying to the clinic for current HIV test						
Partner/spouse	33	10.2 (7.3–14.0)	37	18.8 (13.9–24.9)	70	13.4 (10.8–16.7)
Family/other	57	17.6 (13.8–22.1)	11	5.6 (3.1–9.8)	68	13.1 (10.4–16.2)
No one	234	72.2 (67.1–76.8)	149	75.639–1–81.2)	383	73.5 (69.5–77.1)
Perceived social support						
Medium to high	314	96.9 (94.3–98.3)	189	96.4 (92.7–98.3)	503	96.7 (94.8–98.0)
Low	10	3.1 (1.7–5.7)	7	3.6 (1.7–7.3)	17	3.3 (2.0–5.2)
Depression						
No depression	299	92.6 (89.1–95.0)	175	91.6 (86.7–94.8)	474	92.2 (89.6–94.2)
Major depression	24	7.4 (5.0–10.9)	16	8.4 (5.2–13.3)	40	7.8 (5.8–10.4)
Baseline CD4 count (cells/μL) at testing						
<350	111	33.5 (28.6–38.8)	74	37.4 (30.9–44.4)	185	35.0 (21.0–39.1)
350–500	33	10.0 (7.2–13.7)	20	10.1 (6.6–15.2)	53	10.0 (7.7–12.9)
>500	65	19.6 (15.7–24.3)	9	4.6 (2.4–8.5)	74	14.0 (11.3–17.2)
Missing	122	36.9 (31.8–42.2)	95	48.0 (41.1–55.0)	217	41.0 (36.9–45.3)

### 3.2 Healthcare-seeking behavior and perceived social-support

Overall, 50.2% of study participants had a recent clinic attendance, with a lower proportion of males (38.2%) reporting a recent clinic attendance than females (59.4%). Over a quarter (29.2%) reported testing for HIV for the first time; while 44.3% had their most recent HIV test more than 12 months prior. A total of 33.3% reported having 2 or more sexual partners in the previous 12 months, with over two-thirds not using a condom in their last sexual encounter.

The majority of study participants (95.3%) had medium to high perceptions of current social support. Despite this, 61.0% disclosed their intention to test, and only 26.5% had someone accompanying them to the clinic to test.

Overall 41.0% of study participant had a missing baseline CD4 count. More males (48.0%) had a missing baseline CD4 than females (36.9%), and of those that had a CD4 count 185/312 (59.3) presented with CD4 counts below 350 cell/μl. Those missing baseline CD4 counts were likely to be male, older in age, last tested more than 12 months prior and not have disclosed their intention to test. A total of 80.7% initiated on ART at the testing site up to 6 months after diagnosis.

### 3.3 Self-reported motivators for HIV testing

**Figure 2:**
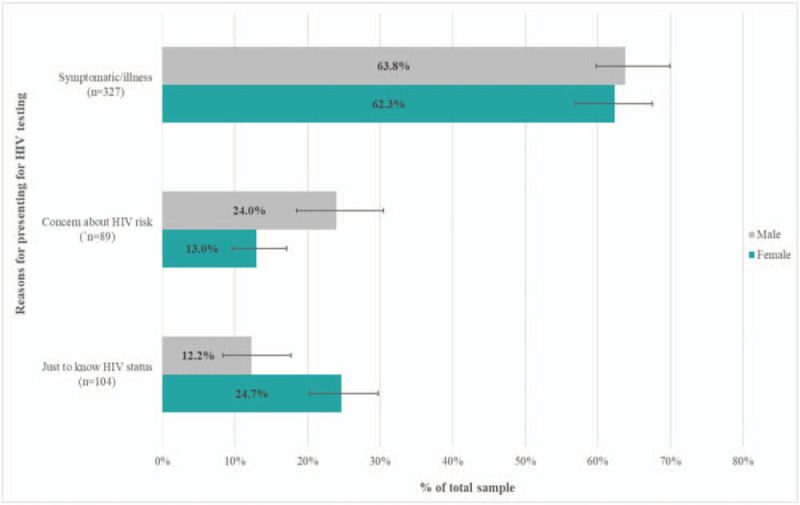
Motivators for seeking HIV testing services.

Among the 520/529 participants with responses, the majority 327/520 (62.9%), reported experiencing symptoms as main motivator for testing for HIV (Fig. 2), 17.1% reported potential HIV exposure as a main reason for testing, and 20.0% just wanted to know their HIV status.

### 3.4 Baseline predictors of self-reported symptoms as a main motivator for HIV testing among newly diagnosed participants

Table 2 presents the crude and adjusted risk ratios of self-reported symptoms being the main motivator for HIV testing. In the adjusted analysis, disclosure of the intention to test (aPR 1.4 for partners/spouse vs family/friend or others, 95% CI: 1.1–1.9; aPR 1.4 for not disclosing vs partners/spouse, 95% CI: 1.1–1.8) were more likely to be motivated to test for HIV by ill-health. Compared to those who tested in the preceding year, first-time testers and those who last tested 12 months prior were more likely to be motivated to test for HIV by symptoms (aPR 1.3 for Never tested for HIV before vs last HIV test < = 12 months ago, 95%CI:1.0–1.6; aPR 1.2 for last HIV test > 12 months ago vs last HIV test < = 12 months ago, 95% CI:1.0–1.4).

**Table 2:** Baseline predictors of symptom related motivators for HIV testing (n = 520).

	Symptomatic	PR	aPR
	n = 327	(95% CI)	(95% CI)
	No. (%)		
Sex			
Female	202 (62.4)	1	1
Male	125 (63.8)	1.0 (0.9–1.2)	0.9 (0.8–1.1)
Age at HIV diagnosis, years			
18–29.99	90 (54.6)	1	1
30–39.99	147 (63.6)	1.2 (0.9–1.4)^†^	1.0 (0.9–1.2)
40+	90 (72.6)	1.3 (1.1–1.6)^∗^	1.2 (0.9–1.4)
Marital status			
Married	47 (63.5)	1	1
In a relationship (living together)	91 (48.7)	0.8 (0.6–0.9)^∗^	0.8 (0.7–1.1)
In a relationship (not living together)	113 (71.5)	1.1 (0.9–1.4)	1.1 (0.8–1.5)
Not in a relationship	75 (75.0)	1.2 (0.9–1.5)	1.1 (0.8–1.4)
English literacy			
I can read very well	171 (58.8)	1	1
I can read somewhat	117 (65.7)	1.1 (0.9–1.3)	1.1 (0.9–1.3)
I cannot read	38 (77.6)	1.3 (1.1–1.6)^∗^	1.1 (0.9–1.3)
Primary source of income			
Paid job, salary or business	214 (64.9)	1	1
Spouse/ partner	50 (53.8)	0.8 (0.7–1.0)^†^	1.0 (0.8–1.2)
Parents/ relatives/ friends/other	62 (65.3)	1.0 (0.9–1.2)	1.0 (0.8–1.2)
Breadwinner of household			
Yes	177 (65.6)	1	
No	149 (60.1)	0.9 (0.8–1.0)	
Access to basic necessities (amenities score)			
Low	17 (56.7)	0.9 (0.7–1.3)	0.8 (0.6–1.1)
Medium	136 (67.7)	1.1 (0.9–1.3)^†^	1.1 (0.9–1.2)
High	166 (60.1)	1	1
Lives with			
Partner/spouse	125 (53.2)	1	1
Family/friends	71 (59.2)	1.1 (0.9–1.3)	0.8 (0.6–1.1)
Alone	92 (78.6)	1.5 (1.3–1.7)^∗∗^	1.0 (0.7–1.4)
Number of child dependants			
None	195 (63.5)	1	
1 child	62 (65.3)	1.0 (0.9–1.2)	
2 or more children	70 (59.3)	0.9 (0.8–1.1)	
Recent clinic attendance (any)			
Never	56 (60.9)	1	
within a year	165 (63.2)	1.0 (0.9–1.3)	
More than a year ago	106 (63.5)	1.0 (0.9–1.3)	
Number of sexual partners in the past 12 months			
None	27 (58.7)	1	
1 Partner	194 (64.7)	1.1 (0.9–1.4)	
>=2 partners	106 (61.6)	1.0 (0.8–1.4)	
Condom use at last sex			
Yes	101 (61.6)	1	
No	226 (63.5)	1.0 (0.9–1.2)	
Last HIV test before current test			
last HIV test <=12 months ago	72 (52.2)	1	1
last HIV test >12 months ago	151 (65.4)	1.3 (1.0–1.5)^∗^	1.2 (1.0–1.4)^∗^
Never tested for HIV before	104 (68.9)	1.3 (1.1–1.6)^∗^	1.3 (1.0–1.6)^∗^
Disclosed intention to test for HIV			
partner/spouse	82 (44.1)	1	1
Family/Friends/Other	93 (71.0)	1.6 (1.3–1.9)^∗∗^	1.4 (1.1–1.9)^∗^
No one	152 (74.9)	1.7 (1.4–2.0)^∗∗^	1.4 (1.1–1.8)^∗^
Person accompanying to the clinic for current HIV test			
Partner/spouse	27 (39.1)	1	1
Family/other	44 (64.7)	1.6 (1.2–2.3)^∗^	1.2 (0.8–1.7)
No one	256 (66.8)	1.7 (1.3–2.3)^∗^	1.2 (0.8–1.6)
Depression			
No depression	298 (63.0)	1	
Major depression	24 (60.0)	1.0 (0.7–1.2)	

## 4 Discussion

This study aimed to explore motivators for HIV testing in the treat-all era and associations between self-reported ill-health as a primary reason for testing and socio-demographic factors. Nearly two-thirds of newly diagnosed HIV infected participants reported symptoms as the main motivator for the latest HIV test, and one third were motivated by HIV risk perception or perceived benefits of testing.^[28]^ These results are comparable to evidence confirming that HIV infected individuals still present for HIV care at an advanced stage of infection in the treat-all era.^[29,30]^

Although HIV testing services have been considerably expanded in South Africa, almost a quarter of our study population reported testing for the first time. This is a higher proportion than previously reported in previous studies, highlighting some of the gaps that still exist in the demand side of HIV testing.^[16]^ A majority of first-time testers were male which aligns with previous evidence demonstrating low uptake of HIV testing among men and poor healthcare-seeking behavior in general.^[16,31]^ Recent studies have highlighted how personal factors and stigma related factors continue to be major barriers to HIV testing as opposed to policy or health system-related factors.^[2,32]^ People still fear a positive HIV test result and the implications that come with it, and it seems many still do not initiate ART as soon as they are diagnosed.^[33,34]^ In our study population, 81% initiated ART within 6 months of diagnosis which is a much higher proportion than the 62.3% current population estimates for South Africa, which includes community based testing.^[12]^ Ill-health may also be a motivator for ART initiation in this cohort.

Of concern is the high proportion of missing baseline CD4 in our cohort. This could be because of CD4 results not being filed or captured in patient's medical records at the site, as we only reviewed medical records on site. It is also possible that blood collection for CD4 counts may not have been done on the day of diagnosis. Either because of logistical issues related to the blood collection service, or patients being in a hurry to leave the clinic, we cannot say with certainty based on our available data.

Almost a fifth of study participants enrolled in the main study presented for HIV testing as new clients but later revealed that they were already aware of their HIV positive status. The majority (72.4%) had a previous HIV test within a year of the current test, pointing to deferred ART uptake possibly related to ART readiness challenges.^[35]^ Repeat testers may also be ART patients who disengaged from care for some time retesting when they re-enter HIV care. Similarly, those who self-transfer would often be required to retest at their referral site before accessing HIV care.^[36]^

We found that the main predictors of symptom-related motivators for HIV testing included HIV testing history, and not disclosed intention for HIV testing. Although perceived social support was widespread, disclosing one's intention to test for HIV could indicate access to actual support. Those already experiencing symptoms may fear disclosing their intention to test for HIV as it may be perceived as already disclosing an HIV positive status. The perceived negative consequences of disclosing the intention to test for HIV in social relationships points to issues of fear and perceived HIV stigma. Encouraging discussions around HIV, and HIV care seeking is important for facilitating social support which has influence on decision-making for HIV status disclosure and engagement in HIV care, even among late testers.^[37–40]^

### 4.1 Limitations

The analysis was limited to those who tested HIV positive and did not assess main motivators for presenting for HIV testing among those that tested HIV negative. Indications are that they may be less motivated to test by ill-health, and more motivated by HIV risk exposure.^[41]^ Future studies assessing main motivators for HIV testing should include all individuals seeking HIV testing service to better understand differences in motivation between those with a positive and negative result. It is also important to increase uptake of routine and repeated HIV testing among this group to facilitate earlier HIV diagnosis.

Additionally, symptoms were generally self-reported and not confirmed by medical records. Here symptoms generally referred to nonspecific ill-health and we did not capture detailed information on conditions that participants presented at testing. Participants were recruited from 4 facilities in Johannesburg which does not represent the full spectrum of health facilities or the HIV positive population in South Africa. Also, the interpretation of these study results is limited to the peri-urban setting from which participants were drawn, and may not necessarily be applicable in rural settings.

## 5 Conclusions

Our findings indicate that newly diagnosed HIV positive patients still enter care as a result of ill-health and not prevention purposes. As individuals may live with HIV for long before the onset of symptoms, increasing early HIV testing remains an essential goal to maximize the benefits of expanded access to ART and reduce opportunities for ongoing transmission.

## Acknowledgments

The authors thank the staff at Bophelong, Rabie Ridge, Mpumelelo, and Diepsloot clinics in Johannesburg.

## Author contributions

**Conceptualization:** Dorina Onoya.

**Data curation:** Idah Mokhele, Tembeka Sineke.

**Formal analysis:** Idah Mokhele.

**Investigation:** Dorina Onoya.

**Project administration:** Idah Mokhele, Tembeka Sineke.

**Resources:** Dorina Onoya.

**Supervision:** Dorina Onoya.

**Writing – original draft:** Idah Mokhele.

**Writing – review & editing:** Idah Mokhele, Tembeka Sineke, Jonas Langa, Dorina Onoya.
